# Predicting the Types of J-Proteins Using Clustered Amino Acids

**DOI:** 10.1155/2014/935719

**Published:** 2014-04-02

**Authors:** Pengmian Feng, Hao Lin, Wei Chen, Yongchun Zuo

**Affiliations:** ^1^School of Public Health, Hebei United University, Tangshan 063000, China; ^2^Key Laboratory for Neuroinformation of Ministry of Education, Center of Bioinformatics, School of Life Science and Technology, University of Electronic Science and Technology of China, Chengdu 610054, China; ^3^Department of Physics, School of Sciences, Center for Genomics and Computational Biology, Hebei United University, Tangshan 063000, China; ^4^The National Research Center for Animal Transgenic Biotechnology, Inner Mongolia University, Hohhot 010021, China

## Abstract

J-proteins are molecular chaperones and present in a wide variety of organisms from prokaryote to eukaryote. Based on their domain organizations, J-proteins can be classified into 4 types, that is, Type I, Type II, Type III, and Type IV. Different types of J-proteins play distinct roles in influencing cancer properties and cell death. Thus, reliably annotating the types of J-proteins is essential to better understand their molecular functions. In the present work, a support vector machine based method was developed to identify the types of J-proteins using the tripeptide composition of reduced amino acid alphabet. In the jackknife cross-validation, the maximum overall accuracy of 94% was achieved on a stringent benchmark dataset. We also analyzed the amino acid compositions by using analysis of variance and found the distinct distributions of amino acids in each family of the J-proteins. To enhance the value of the practical applications of the proposed model, an online web server was developed and can be freely accessed.

## 1. Introduction

J-protein, also known as Hsp40 (heat shock protein 40 kD), is a molecular chaperone protein and is found ubiquitously in both prokaryotes and eukaryotes [1, 2]. J-proteins represent a large family of molecular chaperones and have cooperative functions with Hsp70. Most of the J-proteins contain a “J” domain through which they can interact with and stimulate Hsp70. Based on the structural differences, J-proteins can be classified into four types, that is, Type I, Type II, Type III, and Type IV J-proteins. Type I J-proteins contain an N-terminal J-domain that is separated from the rest of the proteins by a linker “G/F” region (glycine/phenylalanine region) [3, 4]. Distal to G/F region is the zinc-binding cysteine-rich sequence named as “Zinc-finger domain” which distinguishes Type I proteins from other types of J-proteins [4], and Zinc-finger domain is followed by the C-terminal domain [1, 2]. Type II proteins possess all the domains in Type I except the zinc-finger domain [3]. Type III J-proteins contain a C-terminal J-domain but lack both G/F and zinc-finger domains [3]. Type IV, also known as the J-like protein [5], is a group of recently identified proteins that lacks histidine, proline, and aspartate signature motifs in their sequences [4].

By binding Hsp70 and Hsp90, J-proteins play important roles in chaperone cycle regulation and control many physiological functions [4], such as assisting the folding of nascent and damaged proteins, translocation of polypeptides across cellular membranes, and degradation of misfolded proteins [6]. Studies carried out in the past decade have also shown the regulatory roles of J-proteins in cell death. In association with Hsp70, J-proteins not only involve in the folding of caspase-activated DNase which is responsible for the apoptosis-induced DNA fragmentation [7] but also protect the macrophages from nitric-oxide-mediated apoptosis [8]. Gotoh and his colleagues have demonstrated the role of J-protein in the inhibition of Bax translocation to the mitochondria to prevent nitric-oxide-induced cell apoptosis [9]. Kurisu et al. found that MDG1/ERdj4, a member of the human J-protein family, can interact with GRP78/BiP and protect against the cell death induced by endoplasmic reticulum stress in human [10]. The regulation of cell death by J-protein was also reported in plant. Liu and Whitham found that the overexpression of J-protein stimulated the hypersensitive response (HR)-like cell death in soybean [11]. Cancer progressions are also reported to be closely related to J-proteins, but different types of J-proteins play distinct roles [12, 13]. Type I J-protein is tumour promoting, while Type II J-protein acts as tumour suppressors [13]. Therefore, reliably annotating the types of J-proteins is of major importance in order to clarify their distinct biological functions in cell death. However, to the best of our knowledge, there is no computational method for predicting the types of J-proteins.

Keeping these in mind, in the present work, we proposed a model to predict the four functional types of J-proteins based on reduced amino acid alphabet compositions. According to a recent review [14], the rest of the papers are organized as follows: (i) construct a valid benchmark dataset to train and test the predictor; (ii) formulate the samples with an effective mathematical expression that can truly reflect their intrinsic correlation with the target to be predicted; (iii) select a powerful machine learning method to operate the prediction; (iv) perform cross-validation tests to objectively evaluate the anticipated accuracy of the predictor; (v) provide a web server for the prediction method.

## 2. Materials and Methods

### 2.1. Dataset

The sequences of J-protein were taken from the HSPIR database at http://pdslab.biochem.iisc.ernet.in/hspir/, which currently contains 3,901 J-protein sequences [15]. To reduce homologous bias, J-proteins that have ≥40% pairwise sequence identity to each other were removed by using the CD-HIT program [16]. By doing so, we obtained a benchmark dataset containing 1,245 J-proteins that were classified into four types: 63 Type I J-proteins, 53 Type II J-proteins, 1,107 Type III J-proteins, and 22 Type IV J-proteins (Table 1). The benchmark dataset can be freely downloaded from http://lin.uestc.edu.cn/server/iJPred/data.

### 2.2. Reduced Amino Acid Alphabet

Based on the physiochemical properties, the 20 native amino acids can be clustered into a smaller number of representative residues called reduced amino acid alphabet (RAAA) [17–19]. Compared with the traditional amino acid composition, RAAA not only simplifies the complexity of protein system but also improves the ability in finding structurally conserved regions and structural similarity of entire proteins.

Recently, a structural alphabet called protein blocks (PBs) was proposed by de Brevern et al. [20, 21] and has been widely used in computational proteomics as indicated in a review [22]. To aid the design of mutations, Etchebest and his colleagues defined a novel type of RAAA based on PBs [23], where the 20 native amino acids can form five different cluster profiles, that is, CP(13), CP(11), CP(9), CP(8), and CP(5) as shown in Table 2. Ever since it was proposed, RAAA has been widely used for protein family classifications [24–27].

Hence, in the present study, the J-proteins were encoded using the RAAA as formulated by the discrete feature vector** P**:

(1) $$\begin{array}{c} P={\left[\begin{array}{cccccc} f_{1} & f_{2} & \cdots & f_{i} & \cdots & f_{D} \end{array}\right]}^{T}, \end{array}$$

where** T** is the transposing operator and *f*_*i*_ is the occurrence frequency of the *i*th *n*-peptide RAAA and defined as

(2) $$\begin{array}{c} f_{i}=\frac{N_{i}}{L-n+1}, \end{array}$$

where *N*_*i*_ is the number of the *i*th*n*-peptide (*n* = 1, 2, or 3) RAAA in a J-protein with length of *L*. For the different cluster profiles (Table 2) and different values of *n*, the vector dimension (*D*) in (1) will be different. The corresponding dimensions of reduced amino acid (*n* = 1) composition, reduced dipeptide (*n* = 2) composition, and reduced tripeptide (*n* = 3) composition were listed in Table 3.

### 2.3. Support Vector Machine (SVM)

SVM is a powerful and popular method for pattern recognition that has been widely used in the realm of bioinformatics [28–41]. The basic idea of SVM is to transform the data into a high dimensional feature space and then determine the optimal separating hyperplane using a kernel function. To handle a multiclass problem, “one-versus-one (OVO)” and “one-versus-rest (OVR)” methods are generally applied to extend the traditional SVM. For a brief formulation of SVM and how it works, see the papers [28, 29].

In the current study, the LIBSVM 2.84 package [42] was used as an implementation of SVM, which can be downloaded from http://www.csie.ntu.edu.tw/~cjlin/libsvm/. The OVO method was employed for making predictions using the popular radial basis function (RBF). The regularization parameter *C* and the kernel width parameter *γ* were determined via an optimization procedure using a grid search approach using the fivefold cross-validation. In grid research, the search spaces for parameter *C* and *γ* range from 2^15^ to 2^−5^ and from 2^−5^ to 2^−15^ with the steps of 2^−1^ and 2, respectively.

### 2.4. Performance Evaluation

The performance of the method was measured in terms of sensitivity (Sn), specificity (Sp), Matthew's correlation coefficient (MCC), and overall accuracy (OA) defined as follows:

(3) $$\begin{array}{c} \text{Sn}\left(i\right)=\frac{\text{TP}\left(i\right)}{\text{TP}\left(i\right)+\text{FN}\left(i\right)},\\ \text{Sp}\left(i\right)=\frac{\text{TN}\left(i\right)}{\text{TN}\left(i\right)+\text{FP}\left(i\right)},\\ \text{MCC}\left(i\right)=\frac{\text{TP}\left(i\right)\times \text{TN}\left(i\right)-\text{FP}\left(i\right)\times \text{FN}\left(i\right)}{\sqrt{\left[\text{TP}\left(i\right)+\text{FP}\left(i\right)\right]\left[\text{TP}\left(i\right)+\text{FN}\left(i\right)\right]\left[\text{TN}\left(i\right)+\text{FP}\left(i\right)\right]\left[\text{TN}\left(i\right)+\text{FN}\left(i\right)\right]}},\\ \text{OA}=\frac{1}{N}\overset{M}{\underset{i=1}{\sum }}\text{TP}\left(i\right), \end{array}$$

where TP(*i*), TN(*i*), FP(*i*), and FN(*i*) represent true positive, true negative, false positive, and false negative of family *i*; *M* is the number of subsets and equals to 4, while *N* is the number of the total J-proteins in benchmark dataset.

## 3. Results and Discussion

### 3.1. Cross-Validation

Three cross-validation methods, namely, subsampling (or K-fold cross-validation) test, independent dataset test, and jackknife test, are often used to evaluate the quality of a predictor [43]. Among the three methods, the jackknife test is deemed the least arbitrary and most objective as elucidated in [44] and hence has been widely recognized and increasingly adopted by investigators to examine the quality of various predictors [31, 34, 45–50]. Accordingly, the jackknife test was used to examine the performance of the model proposed in the current study. In the jackknife test, each sequence in the training dataset is in turn singled out as an independent test sample and all the rule parameters are calculated without including the one being identified.

The jackknife results obtained by the proposed model on the benchmark dataset based on the five different cluster profiles of the tripeptide (i.e., *n* = 3) case were listed in Table 4. As it can be seen from Table 4, the best success rate of 94.06% was achieved when the predictions were based on CP(8) with a dimension of 512. For comparison, the results of the amino acid (i.e., *n* = 1) and dipeptide (i.e., *n* = 2) cases were also calculated and listed in Table 5, from which we can see that none of them has higher success rates than the case of *n* = 3.

In our previous study [27], the six HSP families were successfully classified by using the dipeptide of RAAA. But for the classification of the J-protein subfamilies in the present work, the best predictive result was obtained by using the tripeptide of RAAA. Hsps belong to the same family share more sequence identity than that of different families [5]; hence we need more suitable parameters to encode the protein sequences as used in the current study.

### 3.2. Comparison with Other Methods

Since there is no published work to predict the types of J-proteins, we could not provide the comparison analysis with existing results to confirm that our presented model is superior to other methods. However, for the purpose of comparison, we compared the results of the present model with that of Random Forest and Naïve Bayes using the same optimal features (the reduced tripeptide compositions based on CP(8)). The results of jackknife test on the benchmark dataset for Random Forest and Naïve Bayes are listed in Table 6. It is shown that the accuracy of SVM is higher than that of Random Forest and Naïve Bayes.

### 3.3. Amino Acids Composition Analysis

**Figure 1:**
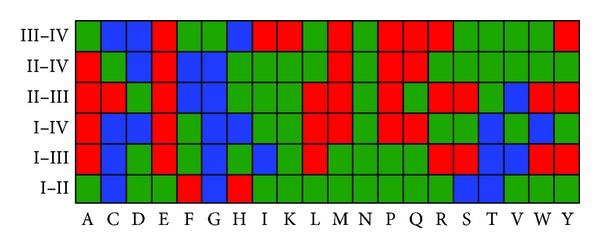
Statistical results to show the divergent distributions of the 20 amino acids among the four (I, II, III, and IV) types of J-proteins. The green boxes indicate that the frequency differences among different types of J-proteins are not significant. The blue boxes indicate that the amino acid is significantly enriched (*P* < 0.05; LSD test) in one type of J-proteins compared with its counterpart. Taking W as an example, the blue box with the coordinate (W, I–IV) indicates that W is enriched in Type I J-proteins compared with Type IV J-proteins. The red boxes indicate that the amino acid is lacking in one type of J-proteins but significantly enriched (*P* < 0.05; LSD-test) in its counterpart. Also taking W as the example, the two red boxes with the coordinates (W, I–III) and (W, II-III) indicate that W is lacking in both Type I and Type II J-proteins compared with Type III J-proteins, respectively.

To provide an overall view, the frequencies of the 20 naive amino acids were compared among the four types of J-proteins using the analysis of variance (ANOVA), and the average amino acid frequency of one type of J-protein with that of another type was further explored and compared using the Fisher's least significant difference (LSD) test. The result is given in Figure 1, where the green boxes indicate that the frequency differences among different types of J-proteins are not significant, while blue and red boxes indicate that the frequency differences are significant (*P* < 0.05; LSD test) among different types of J-proteins (see Figure 1 for more details).

We found that, except Asn (N), the frequencies of all the other 19 amino acids are significantly different among the four types of J-proteins. Compared with other three types, Type I J-proteins are enriched in Cys (C), Gly (G), and Thr (T), Type II J-proteins are enriched in Phe (F), Type III J-proteins are enriched in Ala (A) and Leu (L), while Type IV-J proteins are enriched in Met (M), Gln (Q), Glu (E), and Pro (P) but lack Asp (D) and His (H). The lack of D and H residues in Type IV-J proteins leads to their inability to stimulate ATP hydrolysis [5]. Moreover, according to the binomial distribution [51], we also found the overpresented tripeptides in each family and listed them in Supporting Table S1 in Supplementary Material available online at http://dx.doi.org/10.1155/2014/935719, where the over-presented tripeptides with their confidence levels are provided. These results indicate that the distinct distributions of amino acids in the four types of J-proteins may account for their distinct functions in biological processes.

### 3.4. Web Server Guide

To enhance the value of the practical applications of the proposed model and for the convenience of the vast majority of experimental scientists, an online predictor was developed. The step-by-step guide on how to use it is provided as follows.

**Figure 2:**
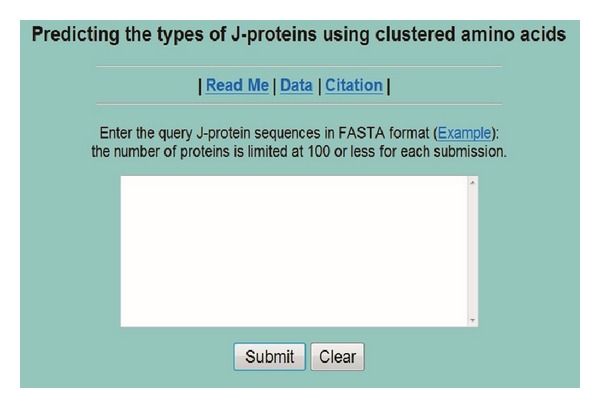
A semiscreenshot to show the top page of the web server. It is available at http://lin.uestc.edu.cn/server/Jpred.

1. Open the web server at http://lin.uestc.edu.cn/server/Jpred and you will see the top page as shown in Figure 2. Click on the* Read Me* button to see a brief introduction about the predictor and the caveat when using it, and click on the* Data* button to download the benchmark datasets used to train and test the predictor. The relevant papers that document the algorithm of the predictor can be found by clicking on the* Citation* button.
2. Either type or copy/paste the query J-protein sequence into the input box at the center of Figure 2. The input protein sequence should be in the FASTA format that can be seen by clicking on the* Example* button right above the input box.
3. Click on the* Submit* button to see the predicted result. For example, if you use the four query J-protein sequences in the* Example* window as the input, after clicking the* Submit* button, you will obtain the results: the outcome for the 1st query sample is “*Type I J-protein*;” the outcome for the 2nd query sample is “*Type II J-protein*;” the outcome for the 3rd query sample is “*Type III J-protein*;” the outcome for the 4th query sample is “*Type IV J-protein*.”

## 4. Conclusion

Cell death is a common phenomenon in developmental processes or in normal physiological conditions and is induced by an array of extra- or intracellular stimuli [7]. However, organisms are equipped with their own physiological defense to cope with environmental stress in order to prevent or induce cell death depending upon the severity of the stress [7]. In mammalian cells, the stress response involves the induction of Hsps, such as Hsp70 and Hsp90. By interacting with J-proteins, these Hsps play pivotal roles in cell death regulations. Since J-proteins act as intermediates, the analysis of J-proteins functions is urgent in order to clarify the regulatory roles of Hsps in cell death.

Based on combination of whole-genome analyses and biochemical evidences, a large number of J-proteins have been identified [6]. However, the exact roles for many of the J-proteins are far from being understood [2, 52]. In order to understand its biological functions, it is highly desirable to know which family a given J-protein belongs to.

By encoding the sequences using the reduced amino acid alphabet information, a predictor was developed to identify the four different families of J-proteins in the present work. To enhance the value of the practical applications of the proposed model and for the convenience of the experimental scientists, an online web server was provided and can be freely accessed at http://lin.uestc.edu.cn/server/Jpred. We hope that the present model will be helpful for scientists who focus on J-proteins and will provide novel insights into the research of cell death.

## Supplementary Material

According to the binomial distribution, we analyzed the distribution of the tripeptides and found the over-presented tripeptides in each J-protein families. Listed in Supporting Table S1 are the over-presented tripeptides with confidence level (=1-p) greater than 99.9%.

## Figures and Tables

**Table 1:** Breakdown of the benchmark dataset used in current study.

Total number	Subfamily	Number
1245	Type I J-protein	63
	Type II J-protein	53
	Type III J-protein	1107
	Type IV J-protein	22

**Table 2:** Scheme for reduced amino acid alphabet based on protein blocks method.

Cluster profiles	Protein blocks method
CP(13)	G-**IV**-**FYW**-A-L-M-E-**QRK**-P-**ND**-**HS**-T-C
CP(11)	G-**IV**-**FYW**-A-**LM**-**EQRK**-P-**ND**-**HS**-T-C
CP(9)	G-**IV**-**FYW**-**ALM**-**EQRK**-P-**ND**-**HS**-**TC**
CP(8)	G-**IV**-**FYW**-**ALM**-**EQRK**-P-**ND**-**HSTC**
CP(5)	G-**IVFYW**-**ALMEQRK**-P-**NDHSTC**

**Table 3:** Feature vector dimension of *n*-peptide composition with different cluster profiles.

*n*-peptide	Cluster profiles				
	CP(13)	CP(11)	CP(9)	CP(8)	CP(5)
*n* = 1	13	11	9	8	5
*n* = 2	169	121	81	64	25
*n* = 3	2197	1331	729	512	125

**Table 4:** Results obtained in identifying J-protein functional types with tripeptide case (*n* = 3).

Subfamily	Metrics	Feature dimension of *n* = 3 for each cluster profile				
		CP(13)	CP(11)	CP(9)	CP(8)	CP(5)
		2197	1331	729	**512**	125
Type I J-protein	Sn	63.49%	74.60%	77.78%	**74.60%**	60.31%
	Sp	99.56%	98.94%	99.11%	**98.76%**	98.93%
	MCC	0.74	0.76	0.79	**0.75**	0.66
Type II J-protein	Sn	37.73%	45.28%	39.62%	**49.06%**	24.53%
	Sp	100%	99.31%	99.39%	**99.05%**	99.56%
	MCC	0.60	0.57	0.53	**0.57**	0.41
Type III J-protein	Sn	99.81%	98.82%	99.09%	**98.56%**	99.19%
	Sp	44.44%	58.78%	55.72%	**62.02%**	40.00%
	MCC	0.63	0.68	0.67	**0.69**	0.56
Type IV J-protein	Sn	0	27.27%	13.64%	**31.81%**	4.54%
	Sp	100.00%	100.00%	100.00%	**100.00%**	100.00%
	MCC	0	0.52	0.37	**0.56**	0.21

OA		93.57%	94.06%	93.98%	**94.06%**	92.36%

**Table:** (a) For the single amino acid case (*n* = 1)

Subfamily	Metrics	Feature dimension of *n* = 1 for each cluster profile					
		CP(20)	CP(13)	CP(11)	CP(9)	CP(8)	CP(5)
		20	13	11	9	8	5
Type I J-protein	Sn	71.42%	65.08%	68.25%	52.38%	50.79%	22.22%
	Sp	98.58%	98.66%	98.66%	98.93%	98.48%	98.03%
	MCC	0.71	0.67	0.69	0.60	0.56	0.26
Type II J-protein	Sn	33.96%	30.19%	33.96%	16.98%	16.98%	15.09%
	Sp	99.82%	99.21%	99.12%	99.47%	99.56%	99.09%
	MCC	0.54	0.42	0.45	0.30	0.31	0.23
Type III J-protein	Sn	98.74%	98.28%	98.10%	99.09%	98.73%	98.19%
	Sp	48.12%	42.86%	45.52%	32.31%	31.54%	17.46%
	MCC	59.71%	0.53	0.54	0.48	0.45	0.26
Type IV J-protein	Sn	4.54%	0	0	0	0	0
	Sp	99.91%	100.00%	100.00%	100.00%	100.00%	100.00%
	MCC	0.15	0.53	0	0	0	0

OA		92.93%	91.97%	92.13%	91.48%	91.08%	89.08%

**Table:** (b) For the dipeptide case (*n* = 2)

Subfamily	Metrics	Feature dimension of *n* = 2 for each cluster profile					
		CP(20)	CP(13)	CP(11)	CP(9)	CP(8)	CP(5)
		400	169	121	81	64	25
Type I J-protein	Sn	74.42%	60.31%	73.02%	60.32%	58.73%	49.20%
	Sp	97.58%	98.59%	98.76%	97.71%	98.32%	97.79%
	MCC	0.75	0.63	0.73	0.58	0.60	0.5
Type II J-protein	Sn	39.76%	45.23%	39.62%	39.62%	35.84%	28.30%
	Sp	94.31%	99.29%	99.48%	99.03%	98.60%	97.99%
	MCC	0.57	0.57	0.54	0.49	0.42	0.31
Type III J-protein	Sn	98.88%	98.10%	98.82%	97.74%	98.01%	97.31%
	Sp	46.37%	50.74%	51.14%	50.79%	48.80%	40.34%
	MCC	60.08%	0.59	0.62	0.57	0.56	0.46
Type IV J-protein	Sn	13.16%	27.27%	0	22.73%	25.00%	9.09%
	Sp	99.91%	99.91%	100.00%	100.00%	100.00%	99.91%
	MCC	0.13	0.48	0	0.47	0.47	0.24

OA		91.47%	92.93%	93.25%	91.97%	92.04%	91.16%

**Table 6:** Comparative result of SVM with other methods for J-protein types classification.

Subfamily	SVM			Random Forest			Naïve Bayes		
	Sn	SP	MCC	Sn	SP	MCC	Sn	SP	MCC
Type I J-protein	74.60%	98.76%	0.75	14.29%	99.55%	0.29	74.60%	92.17%	0.47
Type II J-protein	49.06%	99.05%	0.57	13.33%	99.82%	0.31	54.72%	94.67%	0.39
Type III J-protein	98.56%	62.02%	0.69	99.73%	12.70%	0.31	88.62%	65.83%	0.43
Type IV J-protein	31.81%	100.00%	0.56	4.55%	100.00%	0.21	13.64%	100.00%	0.37

OA	94.06%			89.96%			85.14%		
